# A Survey Study on Knowledge and Attitude Toward the Ethics Committee and Research Ethical Practices Among Researchers From Kuwait

**DOI:** 10.1007/s11948-023-00458-w

**Published:** 2023-10-26

**Authors:** Rashmi Shiju, Smitha Thankachan, Ayesha Akhil, Prem Sharma, Abdullah Bennakhi

**Affiliations:** 1 Office of Regulatory Affairs, Dasman Diabetes Institute, Gulf Road Intersecting, Jassim Al Bahar St. Sharq, Block 3, P.O. Box 1180, Dasman, Kuwait; 2 Special Services Facilities, Dasman Diabetes Institute, Gulf Road Intersecting, Jassim Al Bahar St. Sharq, Block 3, P.O. Box 1180, Dasman, Kuwait City, Kuwait

**Keywords:** Ethics committee, Ethical principles, Informed consent, Misconduct, Knowledge, Awareness

## Abstract

The integrity of research findings and the safety of participants who voluntarily consent to participate in research studies must be assured through ethical approaches. Additionally, ethical guidelines and the ethics committee protect participants from unfair practices by the research team. Therefore, this study aims to assess the knowledge and attitudes toward the ethics committee and research ethical practices among the researchers of a diabetes institute in Kuwait. An anonymous survey was conducted through an online questionnaire using Microsoft Forms. The study had a response rate of 86%. Among the 55 participants in this study, 43 (78%) had ethics training. Researchers involved in more than four research projects were shown to have a much higher awareness of the ethics committee and its role than researchers involved in no projects. Approximately 90% of researchers had training in research ethics and were knowledgeable about informed consent forms and assent, as well as additional protections for vulnerable populations. Ninety-eight percent of respondents were of the view that an ethics committee was necessary. Our study concluded that most of the researchers at the institute were aware of the role of the ethics committee, and ethical principles. However, we recommend that continuous and customized training on research ethics should be provided.

## Introduction

A rising burden of non-communicable diseases is a leading cause of mortality and morbidity in high-income countries (Chen et al., 2018). Several Middle Eastern countries suffer from similar problems (Rahim et al., 2014; Saha & Alleyne, 2018). As a result, over the last decade, this region has experienced an increase in research (Abraham, 2011; Glickman et al., 2009; Silverman et al., 2013). This has improved patient outcomes and advanced medical knowledge through clinical research. Various epidemiological profiles, ethnic groups, technological advancements, and a fully developed infrastructure provide an advantageous environment for clinical research. While clinical research studies are on the rise, Middle Eastern countries are confronted with greater challenges than ever in creating a unified regulatory framework (Nair et al., 2013; Zannad et al., 2019). Furthermore, a lack of awareness among investigators and the absence of nationalized ethics guidelines contribute to this challenge (Sheblaq & Najjar, 2019). Additionally, there were difficulties in finding qualified human resources with effective leadership skills and a lack of standard operating procedures in the research process. To facilitate clinical research in the region, these barriers need to be addressed (Sheblaq & Najjar, 2019). A study that examines research ethics regulations and guidelines in the countries from the Middle East found wide variations and deficiencies when compared with international ones. The Gulf Cooperation Council (GCC) countries have guidelines in place, but a few countries in the Middle Eastern region are still working on them (Alahmad et al., 2012). Creating national regulations on research ethics and mandating that research studies be evaluated by an ethics committee might be an easier way to adhere to research ethics standards. In a study, ethical practices among researchers from the Eastern Mediterranean region who submitted proposals for funding were evaluated based on three criteria: ethical clearance, informed consent form, and confidentiality. According to the results, only 62% of researchers sought ethics committee clearance. In addition, investigators are unaware of the ethical review process, and efforts should be made to increase awareness (Abdur Rab et al., 2008). Few researchers view the ethics committee as a barrier to conducting research and slowing scientific progress due to the limited knowledge about the ethics committee and the ethical review process (Rababa'h et al., 2020). The presence of an ethics committee ensures that all research activities comply with ethics guidelines. The ethics committee must identify and address the area’s leading to misunderstanding among researchers so that standard ethical practices in research are maintained (Brown et al., 2020). A cross-sectional survey conducted in Middle Eastern universities from Egypt, Bahrain and Lebanon associated a lack of ethical awareness with serious research misconduct (Felaefel et al., 2018). To avoid misconduct and improve the quality of research, it is critical to understand the level of ethical awareness among researchers. There have been a few publications from universities in the Middle Eastern region on assessing ethics knowledge and attitudes among researchers (Al Demour et al., 2019; Azakir et al., 2020; El-Dessouky et al., 2011; Rababa'h et al., 2020; Tarboush et al., 2020). According to these studies, the importance of research ethics compliance in clinical research is widely accepted by all researchers and there is a growing awareness of research ethics principles. Studies have highlighted the importance of research ethics to avoid research misconduct and underlined the significance of ethics committee in clinical research. Few studies have also observed an association between the number of research projects handled versus ethics awareness. These studies have concluded that more work is needed to emphasize fundamental aspects of informed consent and the ethics committee (Al Demour et al., 2019; Azakir et al., 2020; El-Dessouky et al., 2011; Rababa'h et al., 2020; Tarboush et al., 2020). However, there have been no published studies in Kuwait analyzing researchers' knowledge and attitudes regarding the ethics committee. The purpose of our survey was to understand the ethical awareness and attitude toward the ethics committee among researchers of our institute. As we know, the quality of research findings and their application are profoundly influenced by the ethical conduct of research and the ethical perception of researchers. The outcome of the study will provide insight into the level of ethical awareness and, consequently, the status of research quality. The results of ethically conducted research can serve both as evidence-based medicine and as a tool for policymakers.

Dasman Diabetes Institute is dedicated to address the diabetes epidemic in Kuwait through focused diabetes research, integrated prevention, training, and education. The institutes’ research aims to translate fundamental/novel research findings into clinical practice and community-based health outcomes. The Institute also continues to expand its national, regional, and international network by enhancing collaborative efforts. Over the past decade, more than 250 research studies have been conducted at the institute, and 100–150 peer-reviewed publications per year.

## Methods

Study Design: During November and December 2019, an anonymous online survey link was sent to all researchers of the institute via an email with an invitation to participate in the study after obtaining the ethical approval. We modified the Microsoft Form template to create the survey link to collect anonymous data.

Study Participants: The study was conducted at a research institute that conducts various research on diabetes and its comorbidities. The institute is a state-of-the-art diabetes research center with world-class research facilities. Additionally, it features an outpatient facility that focuses on treating diabetes patients for medical needs. Currently, the institute undertakes research projects from different specialties, genetics, immunology, epidemiology, molecular biochemistry, clinical trials, animal studies and collaborative research projects. The survey included researchers from various backgrounds, qualifications, and positions.

Study Tool: The purpose of our questionnaire was to obtain information regarding knowledge on ethics and attitude toward the ethics committee among participants in different roles within the research. Using literature (Azakir et al., 2020; El-Dessouky et al., 2011; Tarboush et al., 2020) as a guide, we developed a questionnaire for our study. It has been modified to include country-specific regulations, target responders, and the type of research conducted at our institute. Ethicists, statisticians, and academicians from the institute reviewed, revised, and finalized the questionnaire. The questionnaire was divided into five parts, each having a set of five to thirteen questions.

The first part included respondents' demographics and general characteristics, such as age, gender, years of experience in research, prior ethics training, and the mean number of research projects handled per researcher.

The second part was on knowledge and awareness of the principles and functions of the ethics committee. This section aimed to determine how well respondents understood the ethics committee and its function. Multiple-choice, dichotomous, and five-point Likert-scale questions were used among the question formats.

The third part was on knowledge of research ethics principles. To assess respondents' knowledge, questions were in the form of case study scenarios. The importance and procedures of protocol amendment, informed consent and assent forms, vulnerable populations, and the safety of research participants were assessed through case scenarios.

The fourth part assessed the respondents’ attitude toward the ethics committee using a five-point Likert scale (1, strongly agree; 2, agree; 3, neutral; 4, disagree; and 5, strongly disagree).

The fifth part assessed the respondents' attitudes toward research ethics practices. It had “yes,” “no,” and “neutral” options as choices.

## Statistical Analysis

The data management, analysis, and graphical presentation was done using the computer software ‘Statistical Package for Social Sciences, SPSS version 25.0 (IBM Corp, Armonk, NY, USA). The data from online questionnaire responses were first transferred to Microsoft Excel and then converted to SPSS software for statistical analysis. For categorical variables, such as gender and ethical training, descriptive statistics have been presented as numbers and percentages. The quantitative continuous variables: (age, years of experience, and mean number of projects per researcher) were first ascertained for normal distribution assumption, applying the Kolmogorov–Smirnov test, presented as; mean ± standard deviation or, if skewed, as median and interquartile or range. The Chi-square test or Fisher exact test was applied to find any association or significant difference between categorical variables and presented results as percentages of, “yes” or the “correct” or “positive” responses only. Similarly, the “strongly agree” and “agree” responses on the Likert scale were combined and presented as percent positive responses. In addition, the overall percent response for each subset of the questionnaire was calculated by combining all “yes” or “positive/correct” responses. Researchers were divided into three categories based on their involvement in the number of research projects: “none,” “1 to 3,” and “4 or more.” Using ANOVA or nonparametric Kruskal–Wallis tests, the mean values of continuous variables and overall mean percent responses for each subset were compared between the three categories of research projects involved. A Student *t* test or a Mann–Whitney *U* test was used to compare the overall mean percent ethical training attended or not to different subsets. Statistical significance was defined as a two-tailed probability value “*p*” of < 0.05.

## Results

In this online survey, a total of 55 participants gave their implied consent to participate in the survey with a self-reported response rate of almost 86% (55/64). The questionnaire had forty-four questions that were divided into five groups. These groups assessed the knowledge and attitude toward the ethics committee and research ethical practices.

### Demographics

Table 1 shows the results of the demographic characteristics such as age, gender, overall years of experience, institutional experience, mean number of projects per researcher, and research ethics training among all respondents. Of the total 55 respondents, 43 respondents (78.2%) had research ethics training, while 12 (21.8%) had no sort of previous research ethics training. The number of male respondents was slightly higher than the number of female respondents (54.5% versus 45.5%). Similar results were found with regards to ethics training (male 53.5% and female 46.5%). The mean age of the respondents was 42.8 years (± 10.0SD). Those with ethics training had a significantly lower mean age (41.2 vs 48.5 years; *p* = 0.024) than ethics untrained. The mean total years of research experience was 16.8 years (± 9.1SD) and the years of experience at the institute was 6.4 years (± 3.8SD). Ethics trained respondents did not appear to have a significant relationship with the total years of experience or years of experience at the institute. There was a significant association between ethics trained and the mean number of projects per researcher (*p* = 0.043).

**Table 1:** General characteristics of the respondents with respect to research ethics trained versus untrained

General characteristics	All respondents (*N* = 55)	Prior research ethics training (*N* = 43)	None (*N* = 12)	*p *value*
Gender				
Male	30 (54.5)	23 (53.5)	7 (41.2)	0.766
Female	25 (45.5)	20 (46.5)	5 (58.8)	
Age (years)				
Mean ± SD	42.8 ± 10.0	41.2 ± 8.9	48.5 ± 12.2	0.024
Years of Experience				
Mean ± SD	16.8 ± 9.1	16.0 ± 8.0	19.6 ± 12.2	0.229
Experience at DDI (yrs)				
Mean ± SD	6.4 ± 3.8	6.5 ± 3.6	6.1 ± 4.6	0.984
Median (IQ)	7 (3–9)	8 (4–9)	7 (1–10)	
Mean number of projects per researcher				
None	10 (18.2)	5 (11.6)	5 (41.7)	0.043
1–3	26 (47.3)	23 (53.5)	3 (25.0)	
≥ 4	19 (34.5)	15 (34.9)	4 (33.3)	

^*^General characteristics vs. training: Chi-square or *t* test or Mann–Whitney *U* test

### Knowledge and Awareness of the Ethics Committee and Its Functions

Knowledge and awareness of the functions of the ethics committee among research ethics trained and untrained is represented in Table 2 and among researchers handling a mean number of research projects is presented in Table 3.

**Table 2:** Knowledge and awareness of principles and functions of ethics committee among research ethics trained and untrained respondents (% ‘correct’ responses)

Questions	All respondents (*N* = 55)	Prior research ethics training (*n* = 43)	None (*n* = 12)	*p* value
Do you know when ethical review committee was formed?	41.8	46.5	25.0	0.182
Do you know ERC at DDI is approved by ministry of health?	72.7	76.7	58.3	0.205
Do you know members of Ethical Review Committee?	69.1	69.8	66.7	0.837
Do you know the role of ethical review committee in the research?	94.5	97.7	83.3	0.050
Are you aware of the ethical principles in research?	96.4	100.0	83.3	0.006
Once the protocol or amendments are reviewed in a convened meeting of ERC or by chairman, do you feel ERC respond in a reasonable time frame most of the time?	88.2	87.5	90.9	0.756
Any change in the study protocol needs ERC approval	98.2	97.7	100.0	0.594
How often the ERC meets to review a protocol	78.2	79.1	75.0	0.763
Have you any time felt that the ERC is biased?	92.5	92.9	90.9	0.828
Do you think ERC at DDI reviews research protocol scientifically and ethically	92.5	97.6	75.0	0.009
Mean ± SD	81.1 ± 17.3	83.3 ± 14.3	73.3 ± 24.6	0.079

**Table 3:** Knowledge and awareness of principles and functions of ethics committee among respondents according to the mean number of projects per researcher (% ‘correct’ responses)

Questions	(None)	(1–3)	(≥ 4)	All	*p* value
Do you know when ethical review committee was formed?	50.0	38.5	42.1	41.8	0.820
Do you know ERC at DDI is approved by ministry of health?	50.0	65.4	94.7	72.7	0.019
Do you know members of ethical review committee?	50.0	69.2	78.9	69.1	0.276
Do you know the role of ethical review committee in the research?	80.0	96.2	100.0	94.5	0.070
Are you aware of the ethical principles in research?	80.0	100.0	100.0	96.4	0.009
Once the protocol or amendments are reviewed in a convened meeting of ERC or by chairman, do you feel ERC respond in a reasonable time frame most of the time?	87.5	84.0	94.4	88.2	0.576
Any change in the study protocol needs ERC approval	100.0	96.2	100.0	98.2	0.567
How often the ERC meets to review a protocol	80.0	76.9	78.9	78.2	0.975
Have you any time felt that the ERC is biased?	77.8	92.3	100.0	92.5	0.120
Do you think ERC at DDI reviews research protocol scientifically and ethically	87.5	100.0	84.2	92.5	0.119
Mean ± SD	70.0 ± 28.3	81.5 ± 14.6	86.1 ± 17.3	81.1 ± 17.3	0.050

Researchers trained in ethics were aware of the role of the ethics committee, research ethical principles, and awareness on the method of review of the ethics committee as compared to those untrained in research ethics. (*p* = 0.05, *p* = 0.006 and *p* = 0.009). Similar results were obtained among researchers who handled more than four projects as compared to none. (*p* = 0.070, *p* = 0.009). Overall, researchers handling more than four projects were more knowledgeable and aware of ethical principles and functions of the ethics committee (*p* = 0.050) in comparison to respondents not engaged in projects.

### Knowledge of Ethical Principles

Table 4 and Table 5 represent knowledge of research ethical principles among research ethics trained versus none and among respondents engaged in more than four projects versus none.

**Table 4:** Knowledge of research ethics principles among research ethics trained and untrained respondents (% ‘yes’ or ‘Correct’ responses)

Questions	All respondents (*N* = 55)	Prior research ethics training (*n* = 43)	None (*n* = 12)	*p* value
If you make a substantial amendment to an approved protocol e.g. an additional objective to the approved protocol and the inclusion criteria is changed, when do you think you can start this change	92.7	93.0	91.7	0.873
Nofel is a 11 year-old boy who is attending DDI pediatric clinic along with his parents. You find him eligible for your study based on eligibility criteria fulfillment. His parents signed the consent form and are keen on him participating in the study. But Nofel is crying and upset about the prospect of doing an MRI which is part of the study requirement. Should Nofel be recruited?	90.9	90.7	91.7	0.918
A research is being conducted on 50 pregnant participants to understand gestational diabetes and their newborn babies will be followed until 6 months. Which of the following best describes this situation?	92.7	90.7	100.0	0.273
Which of the following is the guidelines followed for protection of human subjects?	41.8	39.5	50.0	0.516
Which of the following is the function of ERC	94.5	95.3	91.7	0.619
Mean ± SD	92.7 ± 15.7	92.4 ± 16.8	93.7 ± 11.3	0.801

**Table 5:** Knowledge of ethics principles among respondents according to the mean number of projects per researcher (% ‘Correct’ responses)

Questions	(None)	(1–3)	(≥ 4)	All	*p* value
If you make a substantial amendment to an approved protocol e.g. an additional objective to the approved protocol and the inclusion criteria is changed, when do you think you can start this change *(scenario 18)*	80.0	92.3	100.0	92.7	0.142
Nofel is a 11 year-old boy who is attending DDI pediatric clinic along with his parents. You find him eligible for your study based on eligibility criteria fulfillment. His parents signed the consent form and are keen on him participating in the study. But Nofel is crying and upset about the prospect of doing an MRI which is part of the study requirement. Should Nofel be recruited? *(scenario 19)*	90.0	88.5	94.7	90.9	0.765
A research is being conducted on 50 pregnant participants to understand gestational diabetes and their newborn babies will be followed until 6 months. Which of the following best describes this situation? *(scenario 20)*	70.0	100.0	94.7	92.7	0.007
Which of the following is the guidelines followed for protection of human subjects? *(scenario 21)*	20.0	34.6	63.2	41.8	0.048
Which of the following is the function of ERC *(scenario 22)*	100.0	96.2	89.5	94.5	0.437
Mean ± SD	72.0 ± 23.5	82.3 ± 14.2	88.4 ± 10.1	82.5 ± 15.9	0.027

Respondents’ knowledge of ethical principles was assessed using case scenarios that are faced during the execution of the study. All respondents were aware that an amendment to a protocol can be implemented only after obtaining ethical approval, that assent, and parental consent are mandatory requirements when minors are involved in the study and that additional protection must be provided when a pregnant female is involved in the study. While in the case of engagement in projects per respondent, a significant difference was observed in the awareness of ethical principles among respondents engaged in more than four projects versus none (*p* = 0.027).

For question number four, which involves following ethical guidelines, only 41.8% of respondents were familiar with the ethical guidelines and codes. This could be because the questionnaire had a few acronyms, and the options provided made the scenario on guidelines a little confusing.

### Attitude Toward the Ethics Committee

Table 6 and Table 7 represent the attitude toward the ethics committee among research ethics trained versus none and among researchers engaged in more than four projects versus none.

**Table 6:** Attitude toward ethics committee among research ethics trained and untrained respondents (% ‘positive’ responses)

Questions	All respondents (*N* = 55)	Prior research ethics training (*n* = 43)	None (*n* = 12)	*p* value
Ethical review committee (ERC) is an obstacle to research	85.5	88.4	75.0	0.245
There is no requirement of ERC in the research institute	98.2	97.7	100.0	0.594
Research involving human participants directly or indirectly must be reviewed by the ERC	98.2	97.7	100.0	0.594
Clinical trial studies must be reviewed by ERC and approved before trial initiation	98.2	97.7	100.0	0.594
ERC review is not required as the research is scientifically approved	89.1	88.4	91.7	0.746
Members of the ERC have expertise to review research ethics	89.1	88.4	91.7	0.746
Members of the ERC must be trained on ethics periodically	96.4	97.7	91.7	0.326
Concerns or comments of ERC for a reviewed protocol is according to the ethical principles	92.7	93.0	91.7	0.873
Feedback received from ERC for a research protocol is useful	89.1	93.0	75.0	0.077
Mean ± SD	92.9 ± 9.9	93.5 ± 9.5	90.7 ± 11.4	0.391

**Table 7:** Attitude toward ethics committee among respondents according to the mean number of projects per researcher (% ‘positive’ responses)

Questions	(None)	(1–3)	(≥ 4)	All	*p* value
Ethical review committee (ERC) is an obstacle to research	80.0	76.9	100.0	85.5	0.082
There is no requirement of ERC in the research institute	90.0	100.0	100.0	98.2	0.101
Research involving human participants directly or indirectly must be reviewed by the ERC	100.0	96.2	100.0	98.2	0.567
Clinical trial studies must be reviewed by ERC and approved before trial initiation	100.0	96.2	100.0	98.2	0.567
ERC review is not required as the research is scientifically approved	90.0	88.5	89.5	89.1	0.989
Members of the ERC have expertise to review research ethics	100.0	88.5	84.2	89.1	0.427
Members of the ERC must be trained on ethics periodically	90.0	96.2	100.0	96.4	0.391
Concerns or comments of ERC for a reviewed protocol is according to the ethical principles	90.0)	96.2	100.0	92.7	0.650
Feedback received from ERC for a research protocol is useful	90.0)	88.5	89.1	89.1	0.989
Mean ± SD	92.2 ± 11.8	91.9 ± 10.2	94.7 ± 8.6	92.9 ± 9.9	0.621

Figures in rows are for Projects involved-in (None, 1–3, ≥ 4)

Greater than 90% of respondents had a positive attitude toward the ethics committee, irrespective of their training status. Overall, the mean percentage of positive attitude was 92.9. Respondents who attended ethics training had a higher positivity (93.5%) than those who did not (90.7%). In response to a series of nine questions, respondents’ overall attitudes toward the ethics committee ranged from 85.5 to 98.2%. More than half of the respondents considered the ethics committee to be helpful, with 85.5% stating that the ethics committee is supportive. Almost all respondents (98.2%) believed that the ethics committee is a required entity in the research institute and that all research protocols must be reviewed by the ethics committee whether it involves direct or indirect human subject participation. Despite the presence of a scientific review committee, 89.1% believe that the expertise of the ethical committee is also required. Furthermore, 96.4% of respondents agreed that the ethics committee should be trained periodically.

Similar results were found among researchers handling more than four projects versus none (Table 7). The mean percentage of positivity was found to be 92.9%. An overall 92.7% of respondents were satisfied with the comments and concerns received from the ethics committee for the protocol and 89.1% of researchers found that the ethics committee's feedback is constructive and is according to ethical principles. The majority of the respondents (89.1%) agreed that ethics committee members have the expertise to review research ethics in a study protocol.

### Attitude Toward Ethical Research Practice

Table 8 and Table 9 summarize the attitude toward ethical research practices among research ethics trained versus untrained researchers and among researchers handling more than four projects versus none.

**Table 8:** Attitude toward ethical research practices among research ethics trained and untrained respondents. (% ‘positive’ responses)

Questions	All respondents (*N* = 55)	Prior research ethics training (*n* = 43)	None (*n* = 12)	*p* value
Are researchers and teams following the ethical practices?	65.5	65.1	66.7	0.920
Participants of the study should be informed about the entire details of the study including risk and benefits	100.0	100.0	100.0	
Participants can only be enrolled in the study after ethical approval is obtained	100.0	100.0	100.0	
Written informed consent form (ICF) should be obtained from the participants for an interventional study	90.9	95.3	75.0	0.030
Is it mandatory to obtain ethical approval and consent from the patient to use patient's routine clinical test samples for research?	96.4	95.3	100.0	0.447
Confidentiality and privacy of the research participants must be maintained	100.0	100.0	100.0	
Certain information related to study description or risk can be hidden to avoid dropout rate of study participation	85.5	83.7	91.7	0.490
Is it accepted to use the samples of the previous study in another study even if participants have not agreed for future research?	89.1	86.0	100.0	0.170
Is it accepted to modify data in accordance to the outcome of research without harming participants?	69.1	67.4	75.0	0.616
Is it accepted to copy some other's work without giving due credit to the owner?	94.5	93.0	100.0	0.347
Is it accepted not to cite a written source of information that you used to collect data or ideas?	87.3	86.0	91.7	0.605
Is it accepted to use another person's data or ideas without acknowledgement?	98.2	97.7	100.0	0.594
Have you ever tried to manipulate or overrule the decision of ERC?	96.4	97.7	91.7	0.326
Mean ± SD	90.2 ± 10.6	89.8 ± 11.2	91.7 ± 8.3	0.801

**Table 9:** Attitude toward ethical research practices according to the mean number of projects per researcher. (% ‘positive’ responses)

Questions	(None)	(1–3)	(≥ 4)	All	*p* value
Are researchers and teams following the ethical practices?	60.0	69.2	63.2	65.5	0.844
Participants of the study should be informed about the entire details of the study including risk and benefits	100.0	100.0	100.0	100.0	–-
Participants can only be enrolled in the study after ethical approval is obtained	100.0	100.0	100.0	100.0	–-
Written informed consent form (ICF) should be obtained from the participants for an interventional study	80.0	100.0	84.2	90.9	0.079
Is it mandatory to obtain ethical approval and consent from the patient to use patient's routine clinical test samples for research?	100.0	96.2	94.7	96.4	0.769
Confidentiality and privacy of the research participants must be maintained	100.0	100.0	100.0	100.0	–-
Certain information related to study description or risk can be hidden to avoid dropout rate of study participation	90.0	76.9	94.7	85.5	0.222
Is it accepted to use the samples of the previous study in another study even if participants have not agreed for future research?	90.0	84.6	94.7	89.1	0.558
Is it accepted to modify data in accordance to the outcome of research without harming participants?	70.0	61.5	78.9	69.1	0.458
Is it accepted to copy some other's work without giving due credit to the owner?	90.0	92.3	100.0	94.5	0.417
Is it accepted not to cite a written source of information that you used to collect data or ideas?	90.0	76.9	100.0	87.3	0.069
Is it accepted to use another person's data or ideas without acknowledgement?	90.0	100.0	100.0	98.2	0.101
Have you ever tried to manipulate or overrule the decision of ERC?	80.0	100.0	100.0	96.4	0.009
Mean ± SD	87.7 ± 15.0	89.1 ± 10.2	93.1 ± 8.1	90.2 ± 10.6	0.323

Figures in rows are for Projects involved-in (None, 1–3, ≥ 4)

The respondent’s level of positivity was assessed by asking thirteen questions about research practices. The mean percentage of positive attitude among respondents, irrespective of their ethics training status was found to be 90.2%. Almost all respondents agreed that participants should be informed about the risk and benefit of the study in detail, participants’ confidentiality and privacy should be maintained, and that they may be enrolled only after ethical approval. However, significant differences were observed in the opinion of obtaining a written informed consent form in an interventional study among ethics trained versus untrained (*p* = 0.03). 98.2% of the respondents agreed that acknowledgment should be given when using another person’s idea or data. 94.5% of the respondents agreed that due credit to the owner must be given when copying others’ work. Similar results were obtained among respondents engaged in more than four projects versus none. Overall, the mean percentage of positivity was found to be 90.2%.

In our survey, irrespective of ethics training status and the number of projects handled, most of the respondents had a positive attitude toward the research ethics practices of obtaining ethical approval, obtaining informed consent forms, maintaining basic elements of an informed consent process for secondary use of samples, and research integrity.

## Discussion

The results of this survey illustrate the important aspects about the awareness and attitude of researchers toward the ethics committee and research ethical practices in our institute. In the past, many surveys have been conducted to assess the knowledge and attitude toward ethics committee and research ethical practices in other Middle Eastern low- and middle-income countries (Al Demour et al., 2019; Azakir et al., 2020; El-Dessouky et al., 2011; Rababa'h et al., 2020; Tarboush et al., 2020). However, this is the first study from Kuwait which assessed awareness and attitude toward the ethics committee and research ethical practices among researchers.

Our findings revealed a significant association between ethics trained respondents and the mean number of projects per researcher. Similar association was reported between the level of awareness of ethics and prior research experience (Azakir et al., 2020). Ethics-trained researchers and those involved in more research projects had a significantly higher level of awareness of the role and function of the ethics committee and ethical principles. Other authors (Azakir et al., 2020; El-Dessouky et al., 2011) also found the same results. Further, the correct response ranged only from 41.8 to 69.1% when participants were asked about their awareness of the ethics committee's formation, approval by Kuwait's Ministry of Health, and information about committee members. There was a similar trend in overall results among researchers handling one to four projects. Lack of knowledge about committee members was also reported in a qualitative study conducted in Lebanon and Qatar (Makhoul et al., 2014).

The mean age of the researchers was 42 years among our respondents. In our study, the beginners were better trained in research ethics than the experienced researchers. The Collaborative Institutional Training Initiative (CITI) of the National Institute of Health (NIH), is an online training program for research, ethics, compliance, and safety training which the institute has subscribed and renewed annually to cater to its need. This course provides training on Human Subject Research, Conflicts of Interest, Essentials of Public Health Research, Good Clinical Practice (GCP)—Social and Behavioral Research Best Practices for Clinical Research, Social and Behavioral Responsible Conduct of Research, Biomedical research stage II refresher course and GCP for clinical trials with investigational drugs & biologics (ICH focus). In addition to completing the training course, the new staff must attend internal ethical presentations and workshops and submit the certificate to the office of regulatory affairs. A copy of booklet regarding the protection of rights and safety of research participants is also provided to the researcher. There is a difference in the knowledge of ethics between physicians and academic researchers. Physicians understand ethics from the point of view of medical ethics studied in the medical curriculum however, when it comes to research ethics, they lack sufficient knowledge (Al Demour et al., 2019; Azakir et al., 2020).

For research to be conducted ethically, the knowledge of ethical principles is a mandatory requirement among researchers (Resnik, 2012). In our study, irrespective of their ethics training status, 92% of respondents were aware of ethical norms such as the requirement of approaching the ethics committee when there is a substantial amendment to the protocol, requirement of assent, and parental consent when a minor is involved in the study, and requirement of additional protection when a vulnerable population is involved in the study. However, a significant difference was observed in the knowledge of ethical principles between respondents engaged in more than four projects and none. This may be due to the frequent interaction of respondents engaged in more projects with the committee members.

An increase in research projects requires strict monitoring to ensure the quality of studies and compliance with international guidelines. The ethics committee plays an important role in this aspect (Davis, 2018; Ochieng et al., 2013; Pickworth, 2000; Shafiq et al., 2020). Our respondents agreed on the importance of the ethics committee and its need in the institute. Majority of respondents in our study unanimously agreed on the expertise of the ethics committee members, that the protocol reviewed is according to the ethical principles, and that the feedback received from the ethics committee for a research protocol is useful. Overall, the researchers felt that the ethics committee was not an obstacle. On the contrary, a few studies found that the ethics committee is an obstacle to the research process (Rababa'h et al., 2020; Munoli et al., 2017; Tarboush et al., 2020). The respondents felt that the ethics committee needed regular periodic training to enhance and update themselves according to international regulations and guidelines (Al Demour et al., 2019; Rababa'h et al., 2020).

Informed consent is one of the most essential rights of participants in research. Informed consent procedures for research, is a process, not just a document. When a study is conducted in any country, the local participants’ views, law of land and ethnic diversity should be well versed when an informed consent form is explained (Krogstad et al., 2010). Our respondents unanimously agreed that participants should be enrolled only after the study protocol is ethically approved and that the entire informed consent form, including risk and benefit, participant confidentiality, and privacy should be explained to participants in their comprehendible language. However, in our results we found a significant difference between ethics trained versus none about obtaining written informed consent form in an interventional study.

Misconduct is a major concern in the scientific world. An unethical study would not only harm the participants, but also other associated parties. The trust in the data of research relies on honest reporting without plagiarism, fabrication, falsification, and fraud (Fanelli et al., 2015; George & Buyse, 2015). The attitude of our respondents toward responsible conduct of research were assessed through questions on fabrication of data, concealing information from participants to avoid dropouts, and plagiarism. Ninety percent of our respondents agreed that samples should not be used in other studies without the consent of participants. More than 90% of respondents agreed that copying others’ work or ideas without due credit and acknowledgement is a nonacceptable practice. With respect to manipulation of data, around 70% of respondents were against this malpractice. Though 30% of our respondents believed that it is acceptable to fabricate data without harming participants. Most of the respondents in our study showed a positive attitude toward adhering to the research ethical practices.

It is highlighted by many authors that the lack of training contributes to high percentage of the challenges in conducting research studies in Arab countries (Nair et al., 2013; Sheblaq & Najjar, 2019). Our result indicated that the awareness and attitude toward the ethics committee and research ethics practices among our respondents were better. This explains the increasing number of research studies conducted in our institute and becoming one of the centers for clinical trials. According to the study data, our respondents have a fair knowledge and attitude toward research ethics committees and research ethical practices, but there are some gaps in certain areas which require customized and continuous training for physicians and academic researchers.

## Limitation

Ours is an institute with intensive research only on diabetes, hence the number of respondents were relatively small in this survey. Moreover, the survey was conducted at only one institute in Kuwait. While the questionnaire was adapted from published articles, standard validation methods were not used, rather institute experts' review comments were considered. The results did not include researchers who had previously received ethics training before joining our institute.

## Conclusion

This survey aimed to assess the researchers’ knowledge and attitudes about the ethics committee, its role, ethical principles, and research ethical practices. The data obtained from this study is consistent with those obtained from similar studies in other GCC countries. Overall, past studies suggest that while there is a growing awareness of research ethics principles in the Middle East and Northern Africa (MENA) region and GCC countries there is a need for more education and training programs to improve knowledge and implementation of these principles in practice. Additionally, regulatory oversight may be necessary to ensure adherence to research ethics guidelines. The responses obtained were analyzed to find out if there are any differences in the ethical awareness and attitude among the respondents according to their ethics training status and mean projects handled per researcher. There was a significant difference in knowledge and awareness of the ethics committee and its function based on research ethics training and the average number of projects handled per researcher. In our results, regardless of ethics trained or untrained, the presence of ethics committee, and their requirement for ethical review of research involving human participation was much appreciated. Nonetheless, there exists a lacuna in the awareness among researchers which can be bridged by customised and continuous ethics training.
